# Thermoelectric Generators: Alternative Power Supply for Wearable Electrocardiographic Systems

**DOI:** 10.1002/advs.202001362

**Published:** 2020-08-11

**Authors:** Matthew Dargusch, Wei‐Di Liu, Zhi‐Gang Chen

**Affiliations:** ^1^ School of Mechanical and Mining Engineering The University of Queensland Brisbane Queensland 4072 Australia; ^2^ Center for Future Materials University of Southern Queensland Springfield Central Brisbane Queensland 4300 Australia

**Keywords:** electrocardiographic systems, power generators, power supply, thermoelectrics

## Abstract

Research interest in the development of real‐time monitoring of personal health indicators using wearable electrocardiographic systems has intensified in recent years. New advanced thermoelectrics are potentially a key enabling technology that can be used to transform human body heat into power for use in wearable electrographic monitoring devices. This work provides a systematic review of the potential application of thermoelectric generators for use as power sources in wearable electrocardiographic monitoring systems. New strategies on miniaturized rigid thermoelectric modules combined with batteries or supercapacitors can provide adequate power supply for wearable electrocardiographic systems. Flexible thermoelectric generators can also support wearable electrocardiographic systems directly when a heat sink is incorporated into the design in order to enlarge and stabilize the temperature gradient. Recent advances in enhancing the performance of novel fiber/fabric based flexible thermoelectrics has opened up an exciting direction for the development of wearable electrocardiographic systems.

## Graphical abstract

This work overviews that both rigid and flexible thermoelectric power generators, with the advantages of eco‐friendliness and maintenance‐free, are suitable for harvesting electricity from the human body to power up wearable electrocardiographic systems. Moreover, advances in flexible thermoelectric materials will further boost the large‐scale application in electrocardiographic systems.

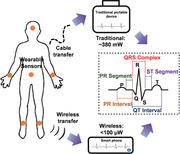

## 1 Introduction

Addressing the health and lifestyle needs of an increasingly ageing population represents one of the great social challenges facing society and this is driving the development of a range of real time health monitoring technologies suitable for easy adoption by elderly citizens.^[^ ^1^, ^2^ ^]^ Electrocardiographic monitoring systems can provide real time heart function signal information including heartbeat periodicity and cardiac muscle depolarization and repolarization characteristics during cardiac cycles which can be used to determine and manage the health conditions of individuals.^[^ ^3^ ^]^

**Figure 1:**
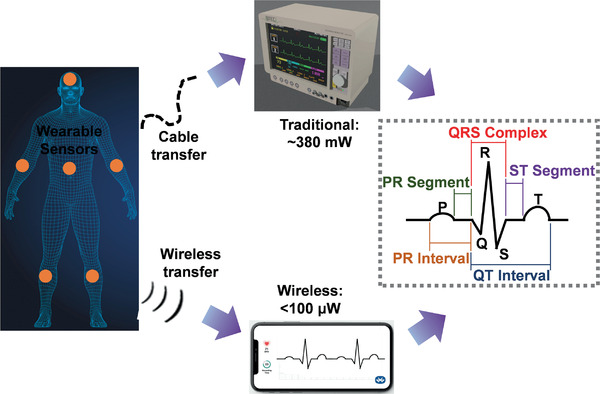
Schematic diagram of signal detection, energy consumption and output signals of traditional and wireless electrocardiographic systems. Reproduced with permission.^[^ ^6^, ^7^ ^]^ Copyright 2020, Verdict Media Limited & Free3D.

Advances in electronic miniaturization and light weight design optimization have resulted in portable and wearable electrocardiographic systems.^[^ ^1^, ^4^, ^5^ ^]^ **Figure** **1** schematically illustrates the signal detection, energy consumption, and output signals of the traditional cable‐connected and wireless wearable electrocardiographic systems.^[^ ^6^, ^7^ ^]^ Both traditional and wireless electrocardiographic systems rely on wearable sensors for signal detection and these devices need to output sufficient signal to deliver real‐time medical information. The incorporation of smart materials and devices can further reduce power supply needs of traditional electrocardiographic systems from the scale of mW to µW.^[^ ^1^, ^6^, ^7^ ^]^

Recent improvements in the performance of thermoelectric materials and design of devices has resulted in the development of a new generation of thermoelectric generators, which can realize eco‐friendly, silent, mechanically simple form of direct energy conversion between heat and electricity suitable for use as power sources for cardiographic health monitoring systems.^[^ ^4^, ^8^, ^9^, ^10^, ^11^, ^12^ ^]^

Both traditional and wireless electrocardiographic systems rely on wearable sensors for signal detection and can effectively output sufficient signals to supply real‐time medical information of individuals. Meanwhile, compared with traditional electrocardiographic systems, combining smart devices with an electrocardiographic system reduces the required power supply from the scale of mW to µW.^[^ ^1^, ^6^, ^7^ ^]^ Thus, thermoelectric generators are suitable for use as power sources for electrocardiographic health monitoring systems.

**Figure 2:**
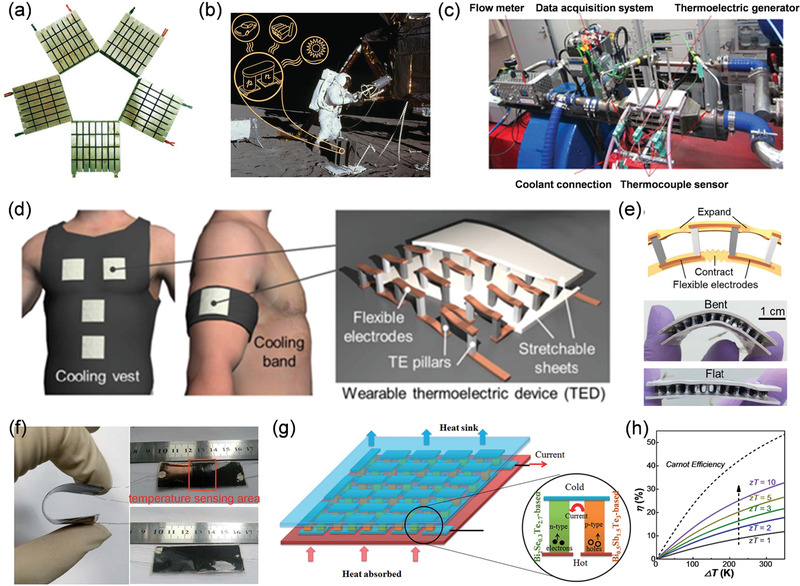
a) Thermoelectric modules. Reproduced with permission.^[^ ^4^ ^]^ Copyright 2016, Nature. b) Thermoelectrics used as radioisotope thermoelectric generators for aerospace missions. Reproduced with permission.^[^ ^13^ ^]^ Copyright 2011, Royal Chemistry Society. c) Thermoelectric devices used for vehicle waste heat recovery. Reproduced with permission.^[^ ^21^ ^]^ Copyright 2018, Elsevier. d) Flexible and wearable thermoelectric modules by employing flexible Cu electrodes and stretchable sheets. Reproduced with permission.^[^ ^30^ ^]^ Copyright 2019, Science. e) Schematic diagram and photograph of the assembled flexible thermoelectric module. Reproduced with permission.^[^ ^30^ ^]^ Copyright 2019, Science. f) Flexible active dual‐parameter sensor for monitoring sensitive temperature and physiological signals. Reproduced with permission.^[^ ^34^ ^]^ Copyright 2019, Royal Society of Chemistry. g) Schematic showing thermoelectric modules. Reproduced with permission.^[^ ^35^ ^]^ Copyright 2018, John Wiley and Sons. h) The maximum energy conversion efficiency (*η*) of a thermoelectric power generator as a function of temperature difference (Δ*T*) under different material dimensionless figure of merit (*zT*) values.

**Figure** **2**a shows typical thermoelectric modules, which are basic units of thermoelectric generators. Initial studies on thermoelectric generators focused on niche applications utilizing specialized high output heat sources such as radioisotopes to generate electrical power supply for aerospace missions (Figure 2b).^[^ ^13^, ^14^, ^15^, ^16^, ^17^ ^]^ Thermoelectric conversion using radioisotope heat sources can generate relatively stable power supply for spacecraft with continuous maintenance‐free heat‐electricity conversion.^[^ ^13^, ^18^, ^19^, ^20^ ^]^ Most previous work also involved the development, application, and evaluation of traditional inorganic thermoelectric materials suitable for use in vehicle waste heat recovery (Figure 2c), where the thermoelectric generators can generate electricity through recycling waste heat from the vehicle engine and waste gas.^[^ ^21^, ^22^, ^23^, ^24^ ^]^ However, the high material cost and insufficient energy conversion efficiency of thermoelectric generators have limited the product development and commercialization.^[^ ^25^, ^26^, ^27^, ^28^, ^29^ ^]^

With the increasing development of multifunctional and miniaturized electronic devices, flexible thermoelectric generators have been developed by using flexible electrodes and substrates. For example, a flexible thermoelectric module can be assembled when thermoelectric legs are combined with flexible Cu electrodes and stretchable sheets (Figure 2d,e)^[^ ^30^, ^31^, ^32^, ^33^ ^]^ Other novel practical applications can also be boosted by the development of highly flexible thermoelectric materials. For example, Zhu et al.^[^ ^34^ ^]^ integrated thermoelectric and piezoelectric materials to form a flexible active dual‐parameter sensor for monitoring a range of physiological signals along with temperature (Figure 2f). Moreover, the improved flexibility of thermoelectric materials and generators can promote their application in electrocardiographic systems. Figure 2g schematically illustrates the structure of a typical thermoelectric module. These modules are composed of p‐n junctions connected in series. In such a module, both the p‐type and n‐type thermoelectric materials are important for the overall energy conversion efficiency.^[^ ^35^, ^36^, ^37^ ^]^ The maximum energy conversion efficiency (*η*) of thermoelectric generators is directly dominated by the material dimensionless figure of merit, *zT* = *S* ^2^ *σ⋅T*/*κ* = *S* ^2^ *σ⋅T*/(*κ*_l_+*κ*_e_), where *S*, *σ*, *T*, *κ*, *κ*_e_, and *κ*_l_ are the Seebeck coefficient, electrical conductivity, temperature, total thermal conductivity, electrical thermal conductivity (*κ*_e_ = *LσT*, and *L* is Lorenz factor^[^ ^11^, ^38^, ^39^, ^40^ ^]^) and lattice thermal conductivity, respectively.^[^ ^27^, ^41^, ^42^, ^43^, ^44^ ^]^ Higher *zT* values lead to higher *η* (Figure 2h).^[^ ^11^, ^45^, ^46^, ^47^ ^]^ The strategy to enlarge *η* is driving the research and design agenda to enhance the thermoelectric power output and minimize the power source requirements in order to provide solutions for electrocardiographic system design and manufacture.^[^ ^48^, ^49^, ^50^, ^51^ ^]^

In this progress report, we comprehensively review the module design strategies that have been adopted to optimize power output of flexible thermoelectric generators for electrocardiographic monitoring systems before highlighting the physical, mechanical, and thermoelectric properties of materials utilized in flexible thermoelectric generators. Finally, we identify promising research directions for the field of thermoelectric powered electrocardiographic systems.

## 2 Thermoelectric Generator Design for Wearable Electrocardiographic Systems

**Figure 3:**
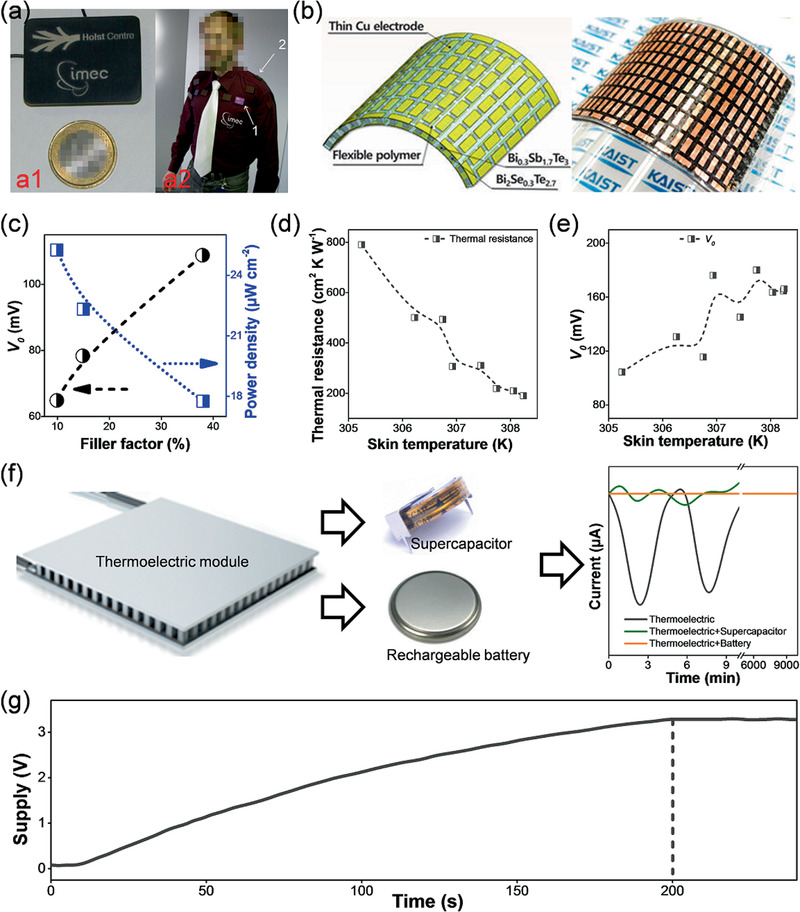
a) The size‐comparison between a thermoelectric module employed for the wireless wearable electrocardiographic system and a one Euro coin (a1) and corresponding thermoelectric shirt (a2), where 1) points at one individual thermoelectric module and 2) is a photovoltaic cell. Reproduced with permission.^[^ ^52^ ^]^ Copyright 2010, Springer. b) Schematic diagram and photograph of the flexible thermoelectric power module assembled with rigid Bi_2_Se_0.3_Te_2.7_ legs, Bi_0.3_Sb_1.7_Te_3_ legs and polymer materials. Reproduced with permission.^[^ ^53^ ^]^ Copyright 2018, American Chemistry Society. c) Power density and open‐circuit voltage (*V*_0_) of as‐assembled flexible thermoelectric module as a function of filler factors.^[^ ^53^ ^]^ d) Thermal resistance and e) *V*_0_ of thermoelectric modules with different skin temperature (at different locations of human body).^[^ ^52^ ^]^ f) A schematic shows the influence of combining thermoelectric modules with batteries in order to stabilize the power output compared with those without batteries and combined with supercapacitors. g) Voltage supply of the charge capacitor integrated in the power management circuit as a function of operation time.^[^ ^53^ ^]^

Traditional rigid thermoelectric modules have been assembled into thermoelectric generators and applied in wearable electrocardiographic systems.^[^ ^52^ ^]^ Additionally, flexible thermoelectric modules with special designs can also be integrated into wearable electrocardiographic systems.^[^ ^53^ ^]^ However, it is essential that thermoelectric system design focuses on achieving good power output stability and density of thermoelectric generators. Traditional rigid thermoelectric modules generally have very limited flexibility (Figure 2a). However recent research has shown that it is now possible to realize new designs that are able to be used in electrocardiographic monitoring applications which are both wearable and provide stable power supplies. (**Figure** **3**a).^[^ ^4^ ^]^

### 2.1 Design Fundamentals for Wearable Thermoelectric Modules

Ceramic plates are widely used in traditional rigid thermoelectric modules and these plates are very inflexible (Figure 2a). However, with appropriate configuration design these rigid modules can be designed to be wearable and render stable power supply for wearable electrocardiographic systems (Figure 3a).^[^ ^4^ ^]^ To realize the flexibility in wearable thermoelectric modules, Kim et al.^[^ ^53^ ^]^ removed the rigid ceramic plates of traditional thermoelectric modules and filled a polymer material with low *κ* of 0.03 W m^−1^ K^−1^ into the space between the thermoelectric legs (Figure 3b). Moreover, the employed Cu electrodes should be as thin as possible in order to ensure the flexibility of the thermoelectric modules. However, thinning Cu electrodes can induce an increased electrical resistance and deteriorate the thermoelectric performance. For this reason, the thickness of the Cu electrodes should be optimized, for example a thickness of 70 µm is suitable for Cu electrodes.^[^ ^53^ ^]^

The filler factor (referring to the ratio between total area of the thermoelectric legs and the whole module) can also affect the performance of the flexible thermoelectric module. Figure 3c shows open‐circuit voltage (*V*_0_) and power density of a designed flexible thermoelectric module as a function of the filler factor. Reducing the filler ratio can lead to reduced *V*_0_ because of the increased number of thermoelectric legs. Additionally, the *V*_0_ increases with increasing filler factor, which is opposite to the trend of power density as a function of filler factor. This may be attributed to the reduced thermal resistance at the ambient‐module interface induced by employing polymers with low *κ* as fillers.^[^ ^53^ ^]^ Furthermore, thinning the thickness of Cu electrodes (from 70 to 35 µm) for the purpose of high flexibility also leads to increased electrical resistance and subsequently slightly reduced power density (≈7%).^[^ ^53^ ^]^

### 2.2 Power Output Stabilization via Additional Electronics

The rigidity of traditional thermoelectric modules results in poor coupling to the human body and therefore the electricity harvested from the human body heat is not stable and cannot be continuously directly used as a stable source to power wearable electrocardiographic systems.^[^ ^52^ ^]^ Figure 3d,e plot the thermal resistance and V_0_ of an individual rigid thermoelectric module as a function of skin temperature at different positions. As is clearly shown, the skin temperature of the human body at different locations has only to change by ≈3 K to dramatically impact the thermal resistance which can vary significantly from ≈800 to ≈200 cm^2^ K W^−1^.^[^ ^52^ ^]^ Simultaneously, *V*_0_ of an individual thermoelectric module can increase from ≈100 to ≈160 mV.^[^ ^52^ ^]^ Therefore, the power output stability is the key challenge when applying traditional rigid thermoelectric generators as wearable power supplies for electrocardiographic systems.

Thermoelectric generators integrated with supercapacitors can act as energy buffers that can increase power output stability, as proposed by Leonov et al.^[^ ^52^ ^]^ The supercapacitors can alleviate the power shortage at the scale of several minutes (Figure 3f). With the power consumption in standby mode as low as ≈1 µW, it is also possible to replace the supercapacitors with rechargeable batteries that can extend the standby time to several days.^[^ ^52^ ^]^ The additional batteries can also reduce the startup time.^[^ ^52^ ^]^ The integration of a charge capacitor in the power management circuit with the start‐up time of ≈200 s (Figure 3g) has fully solved this problem for flexible thermoelectric generators.

While unused, the thermoelectric‐battery hybrid power supply systems still requires ≈1 µW to maintain the system in standby mode.^[^ ^52^ ^]^ However, the self‐discharge behavior of the battery can slowly drain all remaining power and finally lead to power exhaustion.^[^ ^52^ ^]^ Combining the designed power supply system with photovoltaic cells can overcome this challenge.^[^ ^52^ ^]^ Additional photovoltaic cells can continuously power the system instead of a battery by placing the device in light‐available locations.^[^ ^52^ ^]^

### 2.3 Flexible Heat Sink Design for High Power Output

A typical integrated wearable electrocardiographic system can include a wearable electrocardiographic sensor, a voltage booster and a regulator circuit. To power such a system, the power output should reach several mV for the designed thermoelectric generator.^[^ ^53^ ^]^ Normally, traditional wearable thermoelectric generators cannot provide such a power output because of the insufficient temperature difference (Δ*T*) between human skin and the ambient surroundings. To overcome this challenge, Leonov et al.^[^ ^52^ ^]^ replaced the power consuming compact wearable electrocardiographic system with power‐saving wireless sensors and reduced the power consumption to ≈10 µW. Kim et al.^[^ ^53^ ^]^ proposed a flexible heat sink to enlarge the Δ*T* without sacrificing the flexibility.

**Figure 4:**
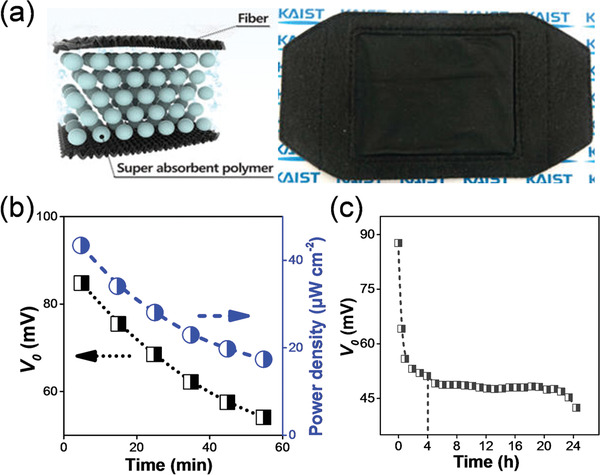
a) Schematic diagram and photograph of a polymer‐based flexible heat sink. Reproduced with permission.^[^ ^53^ ^]^ Copyright 2018, American Chemistry Society. b) Power density and *V*_0_ of the flexible thermoelectric power generator after combining with the flexible polymer‐based heat sink as a function of operating time (<50 min).^[^ ^53^ ^]^ c) *V*_0_ of the flexible thermoelectric power generator after combining with the flexible polymer‐based heat sink as a function of operating time during extended operating period (≈24 h).^[^ ^53^ ^]^

Superabsorbent polymers, which can store large amounts of liquid, such as water, have been employed for flexible heat sink design.^[^ ^53^ ^]^ Superabsorbent polymers can also be combined with an outer fabric to dissipate heat, evaporate water, and maintain Δ*T*. One promising superabsorbent polymer is the commercially available cross‐linked sodium polyacrylate, which contains long‐chained molecules (with multiple repeating units) and electrical charges. After immersion in water, osmotic pressure can push water into the sodium polyacrylate, and the electrical charges can bind with water molecules inside the sodium polyacrylate (**Figure** **4**a).^[^ ^53^ ^]^

It should be noted that one key disadvantage of the polymer‐based flexible heat sink designs is that the evaporation induced water‐loss can lead to a deterioration in power density with continuous operation. As shown in Figure 4b, *V*_0_ and the power density of the flexible thermoelectric generator (after combination with the flexible heat sink) has significantly deteriorated by about ≈40% with continuous operation. During long‐term operation, *V*_0_ can stabilize at ≈50 mV after operating for ≈4 h and remain at this level for ≈18 h (Figure 4c).^[^ ^53^ ^]^ Such a *V*_0_ is superior to that of the comparable flexible thermoelectric device that has been combined with a metal heat sink (stabilized at ≈37 mV after 3 min).^[^ ^53^ ^]^

## 3 Wearable Electrocardiographic Systems

After solving the problems of power output stability and insufficient power generation, wearable thermoelectric modules, and other necessary elements are further integrated into wearable electrocardiographic systems for practical applications.

### 3.1 Wearable Thermoelectric Garment Based on Rigid Thermoelectrics

Combining the rigid thermoelectric module‐based power supply system with a wearable garment such as a shirt requires the thermoelectric modules to be small enough to provide both functionality and comfort for users. The combination of batteries and thermoelectric modules can provide a sustainable solution by achieving the miniaturization goals but maintaining stable and sufficient power supply. Additionally, as the average power production from the human body exceeds the minimum power requirement of a wireless wearable electrocardiographic system by a factor of two, only a small amount of integrated thermoelectric modules are required.^[^ ^52^ ^]^ The reduced module size can make the designed thermoelectric‐battery hybrid power supply system more comfortable when worn by users.

**Figure 5:**
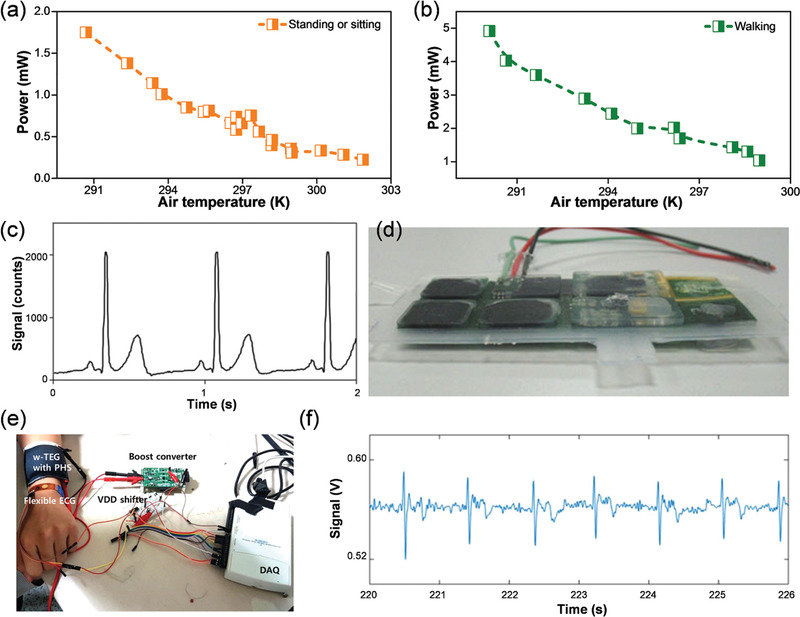
Power output of thermoelectric shirt generated at different ambient temperatures under typical everyday activities such as a) standing or sitting and b) walking.^[^ ^52^ ^]^ c) Double‐sided flex encapsulated thermoelectric modules, wires, and photovoltaic cells. Reproduced with permission.^[^ ^52^ ^]^ Copyright 2010, Springer. d) Electrocardiogram exported by the thermoelectric shirt powered wireless electrocardiographic system.^[^ ^52^ ^]^ Copyright 2010, Springer. e) Photograph of the assembled electrocardiographic system (composed of the wearable thermoelectric generator (w‐TEG) with polymer‐based flexible heat sink (PHS), a wearable power management integrated circuit, a booster converter, a voltage level (VDD) shifter, a data acquisition (DAQ) analyzer and a electrocardiographic module) and corresponding f) Electrocardiographic signal output. Reproduced with permission.^[^ ^53^ ^]^ Copyright 2018, American Chemistry Society.

In order to generate sufficient power (1 mW) for the wearable wireless electrocardiographic system while ensuring comfort for users (by thinning the thermoelectric modules), Leonov et al.^[^ ^52^ ^]^ employed 14 small pieces of thermoelectric modules (Figure 3a1) to assemble a thermoelectric power supplied shirt (Figure 3a2) including a wireless electrocardiographic system. Thermoelectric modules occupied ≈1.5% of the shirt surface area. Under normal office working conditions (temperature ≈23 °C), the thermoelectric garment can generate a power output of 0.8 to 1 mW at ≈1 V while the user is standing or sitting as shown in **Figure** **5**a.^[^ ^52^ ^]^ Figure 5b plots the power output of the thermoelectric shirt when the user walks. As can be seen, the power output has an enhanced gap of 2 or 3 mW. Figure 5c shows the output signal of the thermoelectric garment containing the wireless, powered electrocardiographic monitoring system.^[^ ^52^ ^]^ The wearable thermoelectric shirt can satisfy the power requirements needed by a wireless electrocardiographic system and this configuration can provide an effective health condition monitoring solution for individuals. Furthermore, encapsulating the power supply device with double‐sided flex can protect it through laundering and pressing (Figure 5d).^[^ ^52^ ^]^

### 3.2 Flexible Thermoelectrics‐based Electrocardiographic System

To realize more comfortable electrocardiographic applications, Kim et al.^[^ ^53^ ^]^ integrated the flexible thermoelectric generator and heat sink with other components (Figure 5e). Flexible thermoelectric modules are first integrated with a wearable power management circuit for power management. A voltage level shifter is subsequently introduced to tune the power level from 40–100 mV to 3.8 V. To further tune the voltage to 1 V, which is the working voltage of the electrocardiographic module and the data acquisition buffer, a boost convertor was also connected.^[^ ^53^ ^]^ After deducting the power used by these components, 70 µW of electricity from the thermoelectric power generator can be used to power the electrocardiographic system and the data acquisition buffer. In fact, the electrocardiographic system and the data acquisition buffer need only 15 µW.^[^ ^53^ ^]^ These investigators have shown that a stable and sufficient power supply can be delivered by this type of wearable thermoelectric power generator including presenting typical electrocardiographic signals measured and supplied by the system (Figure 5f).

## 4 Wearable Thermoelectric Materials

**Figure 6:**
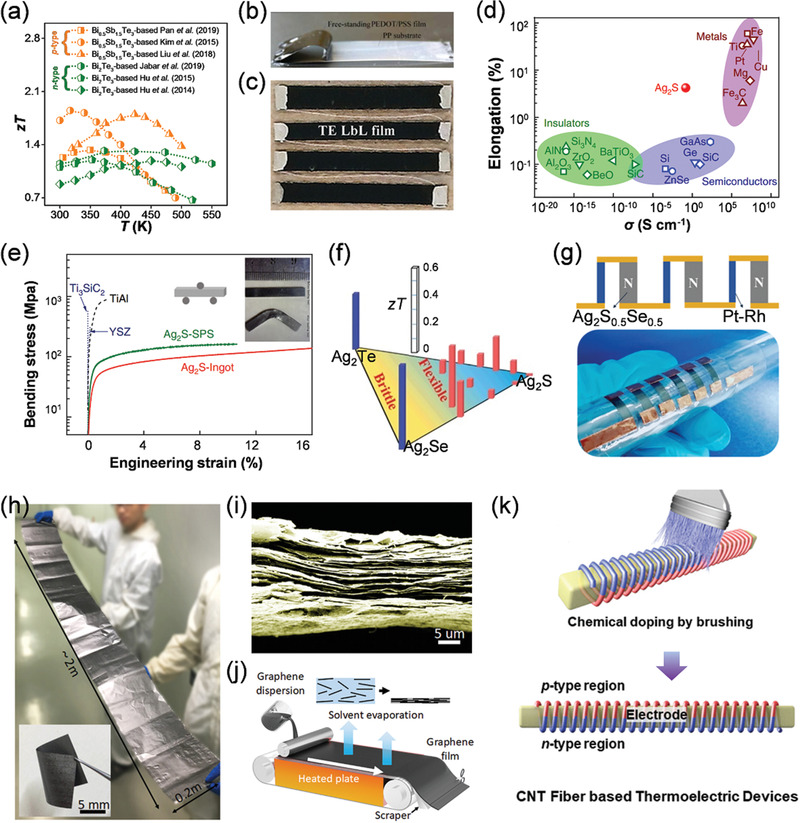
a) Temperature (*T*)‐dependent thermoelectric dimensionless figure of merit (*zT*) values of n‐type Bi_2_Te_3_^[^ ^59^, ^60^, ^61^ ^]^ and p‐type Bi_0.5_Sb_1.5_Te_3_‐based^[^ ^62^, ^63^, ^64^ ^]^ thermoelectric materials. b) Photograph of a flexible free‐standing poly(3,4‐ethylenedioxythiophene)/poly(styrenesulfonate) (PEDOT:PSS) film peeled off from substrate. Reproduced with permission.^[^ ^90^ ^]^ Copyright 2017, Elsevier. c) Photograph of a PEDOT:PSS‐based thermoelectric module patterned on fabrics and connected by Ag wires. Reproduced with permission.^[^ ^79^ ^]^ Copyright 2016, John Wiley and Sons. d) Comparison between elongation of room‐temperature *α*‐Ag_2_S and other materials as a function of electrical conductivity (*σ*). Reproduced with permission.^[^ ^81^ ^]^ Copyright 2018, Nature. e) Bending strain‐stress curves of melt‐synthesized Ag_2_S ingot and spark plasma sintered (SPS‐ed) Ag_2_S pellet in comparison with other materials, including Ti_3_SiC_2_, ceramics yttria‐stabilized zirconia (YSZ) and intermetallic compound TiAl. Reproduced with permission.^[^ ^81^ ^]^ Copyright 2018, Nature. f) The relationship between flexibility and *zT* of Ag_2_(S/Se/Te) system. Reproduced with permission.^[^ ^82^ ^]^ Copyright 2019, The Royal Society of Chemistry. g) A Schematic design of Ag_2_S_0.5_Se_0.5_/Pt–Rh in‐plane module composed of Ag_2_S_0.5_Se_0.5_ as n‐type legs and Pt–Ru wire as p‐type legs, and corresponding optical image of the assembled module. Reproduced with permission.^[^ ^82^ ^]^ Copyright 2019, The Royal Society of Chemistry. h) Photograph and i) Cross‐section scanning electron microscope image of quasi‐industrially produced graphene flakes and corresponding j) Schematic diagram showing the production process. Reproduced with permission.^[^ ^88^ ^]^ Copyright 2019, Elsevier. k) Schematic diagram of the brush‐doping process on wet‐spun prepared carbon nanotubes (CNTs) composing a thermoelectric generator. Reproduced with permission.^[^ ^89^ ^]^ Copyright 2019, Royal Society of Chemistry.

Traditional wearable thermoelectrics used for electrocardiographic systems have been fabricated based on rigid inorganic thermoelectric materials, such as Bi_2_Te_3_,^[^ ^19^, ^29^, ^31^, ^54^ ^]^ PbTe,^[^ ^55^ ^]^ Half‐Heusler,^[^ ^56^ ^]^ and clathrates.^[^ ^57^ ^]^ Within the low‐temperature range (<500 K), the *zT* values of these materials are generally lower than unity.^[^ ^14^, ^27^, ^58^ ^]^ Among them, Bi_2_Te_3_‐^[^ ^31^, ^59^, ^60^, ^61^ ^]^ and Bi_0.5_Sb_1.5_Te_3_‐based^[^ ^62^, ^63^, ^64^ ^]^ materials show room‐temperature *zT* values of >1 (**Figure** **6**a) and have an applicable energy conversion efficiency for wearable electrocardiographic systems.^[^ ^16^, ^27^, ^65^, ^66^ ^]^ However, the rigidity of these materials is a major obstacle because it significantly effects the comfort of the person wearing the system and the coupling of the device to the body heat source.

### 4.1 Flexible Thermoelectric Materials

Recently, with the development of flexible electronics, both organic and inorganic flexible thermoelectric materials are attracting increasing attention. Traditional flexible thermoelectric materials are mostly organic polymers, such as poly(3,4‐ethylenedioxythiophene)/poly(styrenesulfonate) (PEDOT:PSS).^[^ ^20^ ^]^ For example, a free‐standing PEDOT: PSS film is shown Figure 6b. **Table** **1** ^[^ ^67^, ^68^, ^69^, ^70^, ^71^, ^72^, ^73^, ^74^, ^75^, ^76^, ^77^, ^78^, ^79^, ^80^ ^]^ summarizes the room‐temperature thermoelectric performance of state‐of‐the‐art organic thermoelectric materials, including PEDOT:PSS and tetrabutylammonium (TBA). As can be seen, the room‐temperature *zT* value of p‐type PEDOT:PSS can be reported as high as 0.75.^[^ ^73^ ^]^ However, the room‐temperature *zT* value of PEDOT:PSS is below 0.2 in most other reports. Moreover, there have been only limited studies on n‐type flexible organic thermoelectric materials and one of reported room‐temperature *zT* values has been reported to reach 0.23.^[^ ^75^ ^]^ Figure 6c shows a flexible thermoelectric module based on PEDOT:PSS‐based thermoelectric materials which is connected by Ag wires.^[^ ^79^ ^]^ The as‐assembled flexible thermoelectric module can realize a maximum power output of 8.5 nW with a Δ*T* of 5.6 K.^[^ ^79^ ^]^

**Table 1:** Room‐temperature thermoelectric performance of state‐of‐the‐art organic thermoelectric materials, including poly(3,4‐ethylenedioxythiophene)/poly(styrenesulfonate) (PEDOT:PSS) and tetrabutylammonium (TBA)‐based ones. MIm is 1‐methylimidazolium. SWCNT is single‐walled carbon nanotube. TBA is. HA is lindane. CNT is carbon nanotube. PANi is polyaniline. DWNT is double‐walled nanotube

Material	Year	Carrier‐type	*σ* [S cm^−1^]	*S* [µV K^−1^] at 300 K	*S* ^2^ *σ* [µW cm^−1^ K^−2^]	*κ* [W m^−1^ K^−1^]	*zT*	Ref.
PEDOT:PSS+Te (90 wt%)	2019	*p*	200	84	1.42	–	–	^[^ ^67^ ^]^
PEDOT:PSS	2018	*p*	2980	21.9	1.42	0.22	0.190	^[^ ^68^ ^]^
PEDOT:PSS+SiC	2018	*p*	3113	20.3	1.28	0.23	0.17	^[^ ^69^ ^]^
PEDOT:PSS+4:1MIm (32 vol%)	2018	*p*	520	17	0.15	–	–	^[^ ^70^ ^]^
PEDOT:PSS	2018	*p*	2929	17.4	0.89	0.54	0.04	^[^ ^71^ ^]^
PEDOT:PSS+SWCNTs (60 wt%)	2018	*p*	530	44	1.03	0.26	0.12	^[^ ^72^ ^]^
PEDOT:PSS	2018	*p*	1600	68	7.54	0.30	0.75	^[^ ^73^ ^]^
PEDOT:PSS+SWCNTs (74 wt%)	2017	*p*	3800	28	3.00	0.68	0.13	^[^ ^74^ ^]^
TiS_2_(TBA)_0.013_(HA)_0.019_	2017	*n*	450	−140	8.82	1.15	0.23	^[^ ^75^ ^]^
PEDOT:PSS+CNTs (50 wt%)	2017	*p*	2400	49	5.76	–	–	^[^ ^76^ ^]^
PEDOT:PSS	2017	*p*	2170	39.2	3.34	–	–	^[^ ^77^ ^]^
PEDOT:PSS+SnSe (20 wt%)	2016	*p*	320	108	3.86	0.36	0.32	^[^ ^78^ ^]^
PANi/graphene‐PEDOT:PSS/PANi/DWNT‐PEDOT:PSS	2016	*p*	1900	120	27.1	–	–	^[^ ^79^ ^]^
PEDOT:PSS	2013	*p*	885	72	4.56	0.33	0.42	^[^ ^80^ ^]^

Recently, fully inorganic flexible thermoelectric materials have also attracted great interest after the discovery of flexible Ag_2_S semiconductors.^[^ ^81^ ^]^ Figure 6d plots the comparison of elongation of different candidate materials as a function of *σ*. As shown, room‐temperature *α*‐Ag_2_S has intermediate *σ* (demonstrating semiconducting performance) and good elongation (more than ten times higher than traditional semiconductors). Figure 6e shows the bending stress‐strain curves of melt‐synthesized Ag_2_S ingot and spark plasma sintered (SPS) Ag_2_S pellet in comparison with other materials, including Ti_3_SiC_2_, ceramics yttria‐stabilized zirconia (YSZ) and intermetallic compound TiAl.^[^ ^81^ ^]^ Both the directly synthesized ingot and the SPS‐ed pellet of room‐temperature *α*‐Ag_2_S can endure the engineering strain of up to 12% without cracking which is much greater that that achievable by YSZ, Ti_3_SiC_2_ and intermetallic TiAl. Meanwhile, traditional semiconductors or ceramics are very brittle and can tolerate very little plastic bending before cracking. After suitable optimization, the room‐temperature *zT* value of Ag_2_S can approach ≈0.4, which is comparable to those of brittle Ag_2_Te and Ag_2_Se (Figure 6f) systems. Figure 6g shows an assembled module and the corresponding schematic design of a Ag_2_S_0.5_Se_0.5_/Pt–Rh in‐plane module, where the normalized maximum power density can reach as high as 0.08 W m^−1^ under a Δ*T* of 20 K at room‐temperature.^[^ ^82^ ^]^ This is one order of magnitude higher than that produced by organic‐inorganic hybrid thermoelectric materials and prototype flexible modules.^[^ ^82^ ^]^

Carbon‐based materials with high *S* ^2^ *σ* have also recently been reported to show good potential as inorganic flexible thermoelectric materials.^[^ ^83^, ^84^, ^85^, ^86^, ^87^ ^]^ Graphene flakes (Figure 6h,i) with high *S* ^2^ *σ* of 8.4 µW cm^−1^ K^−2^ at room temperature can be quasi‐industrial prepared via film casting (Figure 6j).^[^ ^88^ ^]^ Chemical brushing was also reported to be capable of doping carbon fibers into intrinsic p‐n junctions that could be utilized in a suitable thermoelectric generator assembly, as schematically illustrated in Figure 6k.^[^ ^89^ ^]^ A maximum power density of 259 µW g^−1^ at the Δ*T* of 20 K can be obtained.^[^ ^89^ ^]^

### 4.2 Thermoelectric Coatings on Flexible Fabrics/Fibers

**Figure 7:**
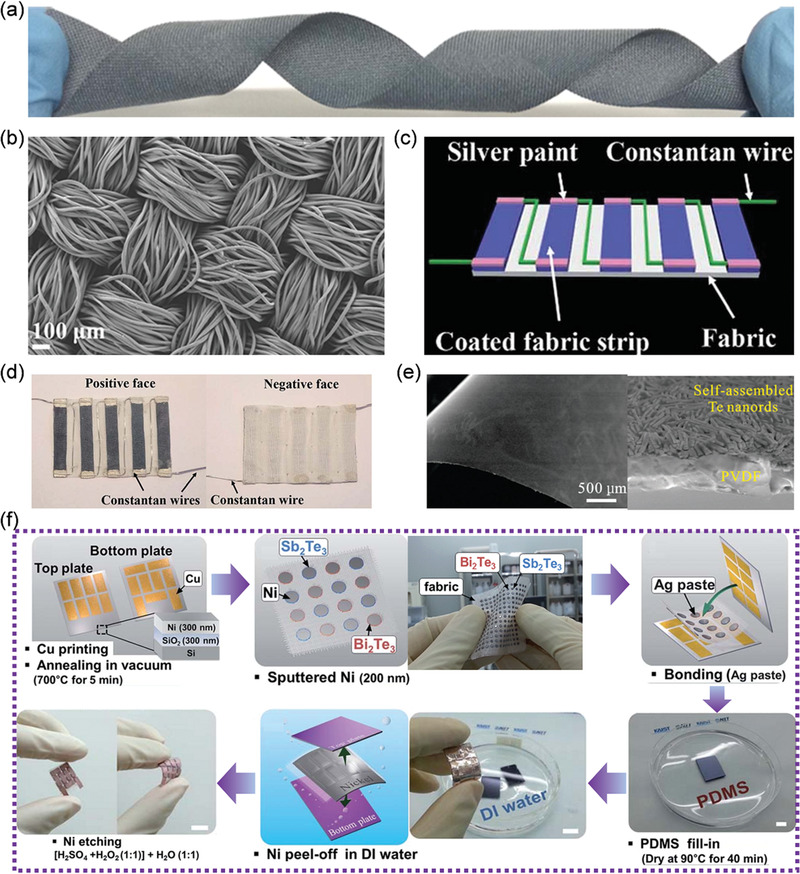
a) Photograph and b) scanning electron microscope (SEM) images of poly(3,4‐ethylenedioxythiophene)/poly(styrenesulfonate) (PEDOT:PSS) coated polyester fabric. Reproduced with permission.^[^ ^96^ ^]^ Copyright 2015, Springer Nature. c) Schematic diagram and d) Photographs of a PEDOT:PSS coated cotton fabric‐based thermoelectric generator composed of five strips that are connected with Constantan wires. Reproduced with permission.^[^ ^98^ ^]^ Copyright 2017, Royal Society of Chemistry. e) SEM image of a self‐assembled Te nanorod‐PVDF composite thermoelectric fabric. Reproduced with permission.^[^ ^100^ ^]^ Copyright 2015, Royal Society of Chemistry. f) Schematic illustration of the fabrication process of a glass‐fiber‐based flexible thermoelectric generator. Reproduced with permission.^[^ ^99^ ^]^ Copyright 2015, Royal Society of Chemistry.

Fiber‐based thermoelectric materials have also been developed by coating thermoelectric materials on fabrics or fibers.^[^ ^91^, ^92^, ^93^, ^94^, ^95^ ^]^ Organic‐based thermoelectric materials, mainly PEDOT:PSS, have been widely coated on polyester fibers,^[^ ^96^ ^]^ composite fibers,^[^ ^97^ ^]^ cotton fabrics,^[^ ^98^ ^]^ silks,^[^ ^91^ ^]^ and yarns,^[^ ^94^ ^]^ etc. The modified fabrics or fibers have been further assembled into prototype flexible thermoelectric modules for *η* assessment and the corresponding performance has been summarized in **Table** **2**, which shows that power outputs are generally at the scale of ≈10 nW.^[^ ^91^, ^94^, ^96^, ^99^ ^]^ **Figure** **7**a,b show a photograph and scanning electron microscope image of a PEDOT:PSS coated polyester fabric, where the voltage can reach several mV at a Δ*T* of ≈50–80 K after proper module design.^[^ ^96^ ^]^ Du et al.^[^ ^98^ ^]^ connected the PEDOT:PSS coated fabric strips with Constantan wires and Ag paints to form a flexible thermoelectric module, as shown in Figure 7c,d. The corresponding *V*_0_ and maximum output electrical power can reach 18.7 mV and 212.6 nW at a Δ*T* of 74.3 K, respectively.^[^ ^98^ ^]^ Additionally, poly(3‐hexylthiophene) (P3HT) and Ag have also been alternatively coated on cotton fabrics for power generation, where the voltage can reach several mV with the power of ≈1 µW.^[^ ^95^ ^]^

**Table 2:** Summary of prototype thermoelectric devices based on wearable thermoelectric fabrics. Δ*T* is the temperature difference between hot and cold sides. *V*_0_ is the open‐circuit voltage. PEDOT:PSS is poly(3,4‐ethylenedioxythiophene)/poly(styrenesulfonate). P3HT is Poly(3‐hexylthiophene‐2,5‐diyl)

Year	Devices	*p*‐legs	*n*‐legs	Δ*T*	*V*_0_	*V*_0_/Δ*T*	Power output	Size	Ref.
2015	Polyester fabric‐based thermoelectric generator (solution coating).	PEDOT:PSS coated fabric strips	Silver wires	75.2 K	4.3 mV	57 µV K^−1^	12.29 nW	Fabric strip: 40 mm × 5 mm	^[^ ^96^ ^]^
2017	Cotton fabric‐ based thermoelectric generator (solution coating).	PEDOT:PSS coated cotton strips	Constantan wires	74.3 K	18.7 mV	253 µV K^−1^	212.6 nW	Fabric strip: 35 mm × 5 mm	^[^ ^98^ ^]^
2018	Sewn‐on thermoelectric coated cotton thread (selective coating).	P3HT	Silver paste	50 K	–	–	1.15 µW	Fabric thickness: 3 mm Cotton thread: length 10 cm, width 1 mm	^[^ ^95^ ^]^
2017	3D structure fabric‐based thermoelectric generator (dip‐coating).	PEDOT:PSS/carbon nanotube composite coated yarns	PEDOT:PSS/carbon nanotube composite coated yarns	66 K	0.8 mV	12 µV K^−1^	2.6 nW	Device size: 6 cm × 6 cm × 7 mm	^[^ ^94^ ^]^
2016	Silk fabric‐ based thermoelectric generator (repeated deposition of thermoelectric paste).	Sb_2_Te_3_	Bi_2_Te_3_	35 K	10 mV	286 µV K^−1^	15 nW	Silk fabric: 4 cm × 8 cm	^[^ ^91^ ^]^
2014	Glass fabric‐ based thermoelectric generator (screen printing).	Sb_2_Te_3_	Bi_2_Te_3_	50 K	90 mV	1800 µV K^−1^	11.4 mW	Device size:15 mm × 20 mm × 0.5 mm	^[^ ^99^ ^]^

Inorganic thermoelectric nanomaterials can also be deposited onto wearable fabrics.^[^ ^100^ ^]^ For example, tellurium nanorods have been deposited onto flexible polyvinylidene fluoride (PVDF) matrix (Figure 7e), where the *S* can reach as high as ≈300 µV K^−1^ at room temperature.^[^ ^100^ ^]^ Moreover, n‐type Ag_2_Te nanocrystal coated nylon has been paired with PEDOT:PSS coated nylon to form a flexible module with a power output of > 5 nW at a Δ*T* of 20 K.^[^ ^101^ ^]^ Similarly, Cu_1.75_Te nanowires have been coated on PVDF.^[^ ^102^ ^]^ Additionally, nano‐sized Bi_2_Te_3_/Sb_2_Te_3_ can also be coated on silk fabrics via vacuum filtration/mechanical pressing/annealing,^[^ ^91^ ^]^ and screen‐printing.^[^ ^93^ ^]^

Proper device design can further boost the thermoelectric performance of the assembled fabric‐based thermoelectric generators. Figure 7f illustrates the fabrication process of a glass‐fiber‐based flexible thermoelectric generator. As shown in Figure 7f, thin and flexible Cu electrodes can be prepared by printing them on Ni(300 nm)/SiO_2_(300 nm)/Si wafers, where the sputtering deposited Ni layer is a sacrificial layer and can separate the completed thermoelectric modules from the Si wafers due to the weak adhesion between Ni and SiO_2_ layers.^[^ ^99^ ^]^ The thin and flexible Cu electrodes need to be further annealed for the purpose of crystallization and densification.^[^ ^99^ ^]^ Bi_2_Te_3_ and Sb_2_Te_3_ thermoelectric dots can be screen‐printed on flexible glass fabrics.^[^ ^99^ ^]^ Cu electrodes and thermoelectric dots can be well‐connected by Ag paste after annealing, where an additional Ni layer can significantly reduce the contact resistance.^[^ ^99^ ^]^ To separate the thermoelectric module from the Si wafer and reduce the energy loss due to air convection, the gaps between the Cu electrodes and thermoelectric dots were further filled with elastic polydimethylsiloxane (PDMS) using a hardening press.^[^ ^99^ ^]^ The liquid‐like PDMS can infiltrate the screen‐printed porous Cu electrodes due to capillary action and form strong connections with the Cu electrodes.^[^ ^99^ ^]^ The assembled thermoelectric generator can be easily peeled off from the Si/SiO_2_ wafers in water due to crack growth at the Ni/SiO_2_ interface.^[^ ^99^ ^]^ The Ni layer can be removed by etching in a mixture of sulfuric acid and hydrogen peroxide. The as‐assembled flexible thermoelectric generator is obtained after this final step.^[^ ^99^ ^]^ Such a generator can produce a power output of 11.4 mW when operating with a Δ*T* of 50 K.^[^ ^99^ ^]^

## 5 Conclusions and Outlook

This review has systematically presented and discussed current research on thermoelectric power generators for application as power sources in wearable electrocardiographic monitoring systems. The output power of thermoelectric power generators can be stabilized and effectively applied to power wearable electrocardiographic systems by proper device design. Employing flexible thermoelectric generators with special polymer‐based flexible heat sinks can further boost the power output. However, current studies of thermoelectric electrocardiographic systems are still focusing on utilizing traditional rigid thermoelectric materials. Recently, promising progress has been reported on the development of flexible and wearable thermoelectric devices, including both flexible thermoelectric materials and fibers/fabrics coated with thermoelectric materials. These highly flexible thermoelectric materials/fibers/fabrics can enable better incorporation of thermoelectric power generators into wearable electrocardiographic systems.

There are three primary focus areas for future research in this field: 1) improved assembly processes and flexible device designs w utilizing recently developed highly flexible thermoelectric materials including fibers/fabrics which can more readily be incorporated into worn products; 2) combining polymer‐based flexible heat sinks with various flexible wearable thermoelectric power generators in order to facilitate higher power output; 3) developing highly processable polymer‐based thermoelectric generators that can be easily coated.

In summary, the incorporation of new flexible thermoelectric materials along with proper design strategies will provide the pathway to deliver sufficient power from flexible thermoelectric power generators to support the operation of wearable electrocardiographic monitoring systems. Further development of flexible thermoelectric materials/fibers/fabrics will also produce more wearable and durable products.

## Conflict of Interest

The authors declare no conflict of interest.
